# Protective effect of silymarin on tacrolimus-induced kidney and liver toxicity

**DOI:** 10.1186/s12906-022-03803-x

**Published:** 2022-12-13

**Authors:** Funda Terzi, Mustafa Kemal Ciftci

**Affiliations:** 1 Faculty of Veterinary Medicine, Department of Pathology, Kastamonu University, 37150 Kastamonu, Turkey; 2 Faculty of Dentistry, Department of Basic Science, Istanbul Gelişim University, 34295 Istanbul, Turkey

**Keywords:** Silymarin, Tacrolimus, Nephrotoxicity, Hepatotoxicity, Histopathology, TUNEL, Rat

## Abstract

**Background:**

Tacrolimus (FK506) is an immunosuppressive agent and has toxic side effects such as nephrotoxicity, hepatotoxicity, and neurotoxicity. In our study, we aimed to investigate the protective effect of silymarin on renal and hepatic toxicity considered to be tacrolimus related.

**Methods:**

In this 6-week experimental study, 46 eight-week-old healthy male rats were used. The groups comprised the Control (healthy rats, n = 6), Tac (tacrolimus 1 mg/kg, n = 8), silymarin 100 mg/kg (SLI 100 mg/kg n = 8), Tac + SLI 100 (tacrolimus 1 mg/kg + SLI 100 n = 8), SLI 200 (SLI 200 mg/kg n = 8), and Tac + SLI 200 (tacrolimus 1 mg/kg + SLI 200 mg/kg n = 8). After 6 weeks, all rats were sacrificed, and the tissue follow-up procedure was performed for kidney and liver tissues, histopathology, and in situ TUNEL analysis. Blood samples were analyzed for the total antioxidant capacity (TAC), total oxidant capacity (TOC), alanine aminotransferase (ALT), aspartate aminotransferase (AST), gamma-glutamyl transferase (GGT), albumin, total bilirubin, creatine.

**Results:**

Histopathological findings of kidney and liver tissue of rats were determined to increase statistically in Tac group compared to SLI 1 00 and SLI 200 groups (*P* < 0.05). In addition, the Tac + SLI 100 and Tac + SLI 200 groups were found to be statistically similar to the Control group (*P* > 0.05). The in situ TUNEL method showed that the tacrolimus increased apoptosis while the silymarin decreased it. TOC levels increased statistically in Tac groups compared to silymarin-treated groups (*P* < 0.05). Although the TAC level was not statistically significant among the experimental groups (*P* > 0.05), the lowest was measured in the Tac group. The ALT, AST, GGT, total bilirubin, and creatine values were higher in the Tac group than in the silymarin groups (*P* < 0.05). There was no statistically significant difference between the groups with regard to the albumin level (*P* > 0.05).

**Conclusion:**

In our study, we determined that tacrolimus caused damage to kidney and liver tissue. Histopathological, biochemical and apoptotic findings show that silymarin has a protective effect against nephrotoxicity and hepatotoxicity caused by tacrolimus.

## Introduction

Tacrolimus (FK506) and cyclosporine are immunosuppressive agents commonly used to prevent transplant rejection and treat some autoimmune diseases [1]. Serious side effects, such as nephrotoxicity, hepatotoxicity, neurotoxicity, glucose intolerance, gastrointestinal toxicity, and post-transplant lymphoproliferative disease, have been reported following tacrolimus administration [2]. Its side effects include nephrotoxicity and hepatotoxicity, which remain a serious problem.

Tacrolimus binds to the binding protein (FKBP) in T cells, forming a new complex molecule (FK506/FKBP). The FK506/FKBP molecule also inhibits calcineurin by binding to calcineurin, which is involved in dephosphorylation, kinase, and phosphatase activity and protein expression in cells [3–5]. With the blocking of calcineurin by tacrolimus, the production of T cell-derived cytokines, such as IL-2–IL-7, interferon-γ, and tumor necrosis factor alpha (TNF-α), the granulocyte macrophage colony stimulating factor is stopped [6, 7]. Thus, tacrolimus suppresses the activity of T lymphocytes and prevents tissue rejection [8, 9]. Tacrolimus undergoes significant pre-systemic metabolism by gastrointestinal CYP 3A4 and P-glycoprotein, which limits its oral bioavailability [10]. In the systemic circulation, approximately 99% of tacrolimus is bound to erythrocytes with α-acid glycoprotein and albumin [11]. Systemically available tacrolimus is metabolized by hepatic CYP3A4/5. - - Tacrolimus can lead to the production of reactive oxygen species (ROS) (hydroxyl radicals (OH¯), superoxide anions (O_2_¯) and hydrogen peroxide (H_2_O_2_)) and the induction of apoptotic cell death due to impaired mitochondrial and T cell functions [12, 13]. Superoxide is converted to H_2_O_2_ and oxygen by the antioxidant enzyme superoxide dismutase (SOD). Hydrogen peroxide can be converted to water and oxygen by enzymes such as catalase, peroxidase and glutathione peroxidase (GPX), or it can react with metal ions (Fenton reaction) to become more reactive hydroxyl radicals [14, 15]. Total oxidant level (TOS) and total antioxidant level (TAS) measurements are preferred in studies of serum and/or plasma concentrations of free oxygen radicals and antioxidants.

Tacrolimus activates the renin-angiotensin system, prostaglandins, and endothelin I in the kidneys, creating vasoconstriction in afferent arterioles, and thus hypoxia-related changes occur in the tubular epithelium in the tissues [3]. Blood urea nitrogen (BUN) and serum creatinine are important markers of kidney damage. Tacrolimus treatment increases blood urea nitrogen (BUN) and serum creatinine levels and decreases endogenous creatinine clearance, indicating decreased renal function [16, 17]. In human studies, adverse effects of tacrolimus at appropriate doses on renal hemodynamics and blood pressure have not been determined in healthy individuals [18]. In kidney transplant patients, cyclosporine has been reported to cause variable severity of phasic hypoperfusion in small to medium-sized intrarenal arteries immediately after dosing, and tacrolimus treatment did not alter renal cortical perfusion [19]. Cyclosporine caused a 49% reduction in renal allograft microperfusion, whereas tacrolimus intake did not cause a significant reduction in renal allograft perfusion [20]. Tacrolimus was more beneficial for clinical outcomes than cyclosporine in preventing graft loss and immunosuppression after primary liver transplantations in adults [21, 22]. Clinical biochemistry and histopathology are important criteria for determining liver changes. Tacrolimus causes hyperbilirubinemia with an increase in liver enzymes ALT, AST, GGT levels [23–25]. In liver and kidney transplant patients and in experimental studies, tacrolimus has been found to have hepatotoxic effects due to liver enzyme levels and histopathological changes in biopsy liver samples [23–26].

Silymarin is an extract obtained from the seeds of *Silybum marianum* (L.) Gaertn (milk thistle) [27]. This mixture contains 65–80% flavonolignans (silybin A, silybin B, isosilybin A, isosilybin B, silychristin, and silydianin) with small amounts of flavonoids and polyphenolic compounds [28–30]. Most of the beneficial effects of silymarin are attributed to its predominant and primary active ingredient, silybin, which is present in proportions of roughly 30% [30, 31].Silybin is a pure, chemically defined substance [32]. Silymarin exerts its antioxidative effect by removing free oxygen radicals and inhibiting lipid peroxidation [33–35]. In addition, silymarin has biological functions, such as being antiinflammatory, antifibrotic, anti-lipid-peroxidative, regulating the permeability of cell membranes and regeneration of hepatocytes [36]. Silymarin is used as a liver protector against alcohol, various chemical substances, mushroom poisoning, snake and insect bites, and therapeutically in liver and gall bladder diseases, such as hepatitis, cirrhosis, and jaundice [4, 29]. Furthermore, in experimental studies, it has been reported that silymarin has a hepatoprotective effect against paracetamol, carbon tetrachloride, zearalenone, valproic acid, and diclofenac-induced liver toxicity [37–40]. The antioxidant Silymarin was found to be nephroprotective against drug nephrotoxicity models in rats. Silybin was reported to be effective in preventing the damage of free oxygen radicals on tubular epithelium, during cold ischemia and the reperfusion period in renal transplantations [32]. Additionally, silybin and/or silymarin were effective against renal damage caused by paracetamol, cisplatin, vincristine, vancomycin, colistin, and diclofenac [41–44]. There are no studies using silymarin against tacrolimus hepatotoxicity and nephroxicity.

In this context, the aims of the present study were to investigate the protective effects of silymarin oxidative stress, nephrotoxicity, and hepatotoxicity with histopathological, in situ TUNEL, and biochemical methods in rats treated with the intraperitoneal administration of tacrolimus.

## Materials and methods

### Animals

A total of 46 Wistar albino female rats, aged 8–10 weeks and weighing 200–220 g, were purchased from the Selcuk University Experimental Medicine Research and Application Center. The experiments were conducted according to European, national, and institutional guidelines for animal welfare and were approved by the local ethics committee of Selçuk University (E.50,144 − 2015/49). The rats were kept in at temperature ranging between 19 and 22 °C, 60–70% humidity, with a standard light/dark photoperiod of 12:12. The rats were fed standard rat aliment and water was provided ad libitum.

### Experimental protocol

The rats were randomly selected and divided into 6 groups: CN (healthy rats, n = 6), Tac (tacrolimus 1 mg/kg/day, n = 8), SLI 100 (silymarin 100 mg/kg/day, n = 8), Tac + SLI 100 (tacrolimus 1 mg/kg/day and silymarin 100 mg/kg/day, n = 8), SLI 200 (silymarin 200 mg/kg/day, n = 8), Tac + SLI 200 (tacrolimus 1 mg/kg/day and silymarin 200 mg/kg/day). In this study, intraperitoneal (IP) tacrolimus (1 mg/kg/day; Astellas Ireland Co. Ltd., Ireland) and silymarin (100 mg/kg/day and 200 mg/kg/day; Cat. No: S0292, Sigma-Aldrich, St. Louis, MO, USA) were administered orally on a daily basis for 6 weeks. At the end of the study, blood samples were taken from the tail veins of the rats for biochemical analysis, and stored at − 20 °C for biochemical analysis. The rats were euthanized by thiopental anesthesia and necropsy was performed. Following necropsy, the liver and kidney tissues were examined grossly and fixed in buffered neutral formalin.

### Histopathological analysis

Routine histopathological procedures were performed and 5-micron-thick sections were taken using a microtome. These sections were stained with hematoxylin-eosin (H&E) and examined under light microscopy (Olympus BX51, Tokyo, Japan) after being adhered to slides. The changes observed in the liver and kidney tissue of the H&E-stained sections were scored between 1 and 4. According to these, the histopathologic changes in the kidney comprised: + (1); the glomerular and tubular structures were normal, ++ (2); mild swelling and hyperemia in the glomeruli, and swelling of the tubulus epithelium, +++ (3); moderate swelling and hyperemia in the glomeruli, and hydropic degeneration in the tubulus epithelium, ++++ (4); severe swelling and hyperemia in the glomeruli, hydropic degeneration of the tubular epithelium, dilatation of the lumens by necrosis and desquamation, and proteinrich fluid, and hyperemia in the interstitial tissue. In the liver scoring: + (1); the hepatocytes and sinusoids were normal, ++ (2); mild swelling in hepatocytes, and narrowing of the sinusoids, +++ (3); moderate swelling and hydropic degeneration in the hepatocytes, and a significant narrowing in sinusoid, ++++ (4); severe hydropic degeneration in the hepatocytes, necrosis and vacuolar formation in some of the hepatocytes, severe narrowing of the sinusoids, and in some. Sections were examined blindly by two pathologists.

### In situ TUNEL analysis

Terminal deoxynucleotidyl transferase-end labeling (TUNEL) staining was conducted using the TdT-FragelTM DNA Fragmentation Detection Kit (Cat. No. QIA33, Calbiochem, USA) In brief, five-micron-thick tissue sections were deparaffinized in the xylene and then rehydrated gradually in an ethanol series (96%, 80%, 70%). Subsequently, the proteinase K was incubated (10 mM TRIS (pH 8), 1:100) at room temperature. Endogenous peroxidase activity was inhibited by incubation with 3% hydrogen peroxide. The sections were incubated with 1X TdT equilibration buffer (pH 6.6, 100 µl)and TdT labelling was then performed in a humidified atmosphere. Then, sections were incubated with stop solution (0.5 M EDTA, pH 8, 100 µl). Each cross-section was treated with blocking buffer (4% BSA in PBS, 100 µL) and then with 1X conjugate (50 X conjugate: diluted 1:50 in blocking buffer). The section labeling was conducted using DAB (3.3 ids diaminobenzidine) solution. Finally, the slides were counterstained with methylene blue solution and dehydrated by passing them through alcohol (96%, 100%), cleared, and mounted closed with Entellan.

### Biochemical analysis

In the biochemical analysis, the AST, ALT, total bilirubin, albumin, and creatine levels were measured in a Roche modular device using the enzymatic colorimetric method.

### Determination of total antioxidant capacity (TAC)

Blood serum total antioxidative capacity (TAC) levels were determined with a spectrophotometric kit (Rel Assay Diagnostics, Gaziantep, Turkey). The novel automated method is based on the bleaching of the characteristic color of a more stable 2,2′-azino-bis[3-ethylbenzothiazoline-6-sulfonic acid] (ABTS) radical cation by antioxidants [45]. Antioxidant molecules in the sample reduce the blue-green ABTS radical to the colorless ABTS form and cause the loss of its characteristic color. After the serum sample was added, the oxidative reactions were initiated by the hydroxyl radicals in the reaction mixture and were suppressed by the antioxidant components of the serum. The results were expressed as mmol Trolox equiv./L.

### Determination of total oxidant capacity (TOC)

The total oxidant level (TOC) was determined by the spectrophotometric method in accordance with the Rel Assay Diagnostics (Turkey) kit procedure. The TOC levels were measured by a novel method based on automated colorimetric measurement developed by O Erel [46]. In the sample, oxidants transform the ferrous ion–o-dianisidine complex into the ferric ion. The ferric ion produces a colored complex with xylenol orange in an acidic medium. The color intensity is associated with the amount of oxidant molecules present in the sample. Hydrogen peroxide was used for the assay calibration and the analysis results were presented as µmol H_2_O_2_ equivalent/L.

#### Statistical analysis

IBM SPSS Statistics for Windows 22.0 (IBM Corp., Armonk, NY, USA) was used for the analysis of the obtained data. One-way analysis of variance (ANOVA) was used for the intergroup comparison of biochemical parameters. The Duncan test is used for the multiple comparisons (post hoc) analysis. To compare the histopathologic injury scores among the different groups, the Kruskal-Wallis test was applied. For parameters with *P* < 0.05, pair-wise comparisons were used using the Mann–Whitney U-test. A Bonferroni correction was performed, and *P*-values under 0.05 were considered significant.The descriptive values of variables were expressed as the mean ± SD.

## Results

### Histopathological results

**Fig. 1:**
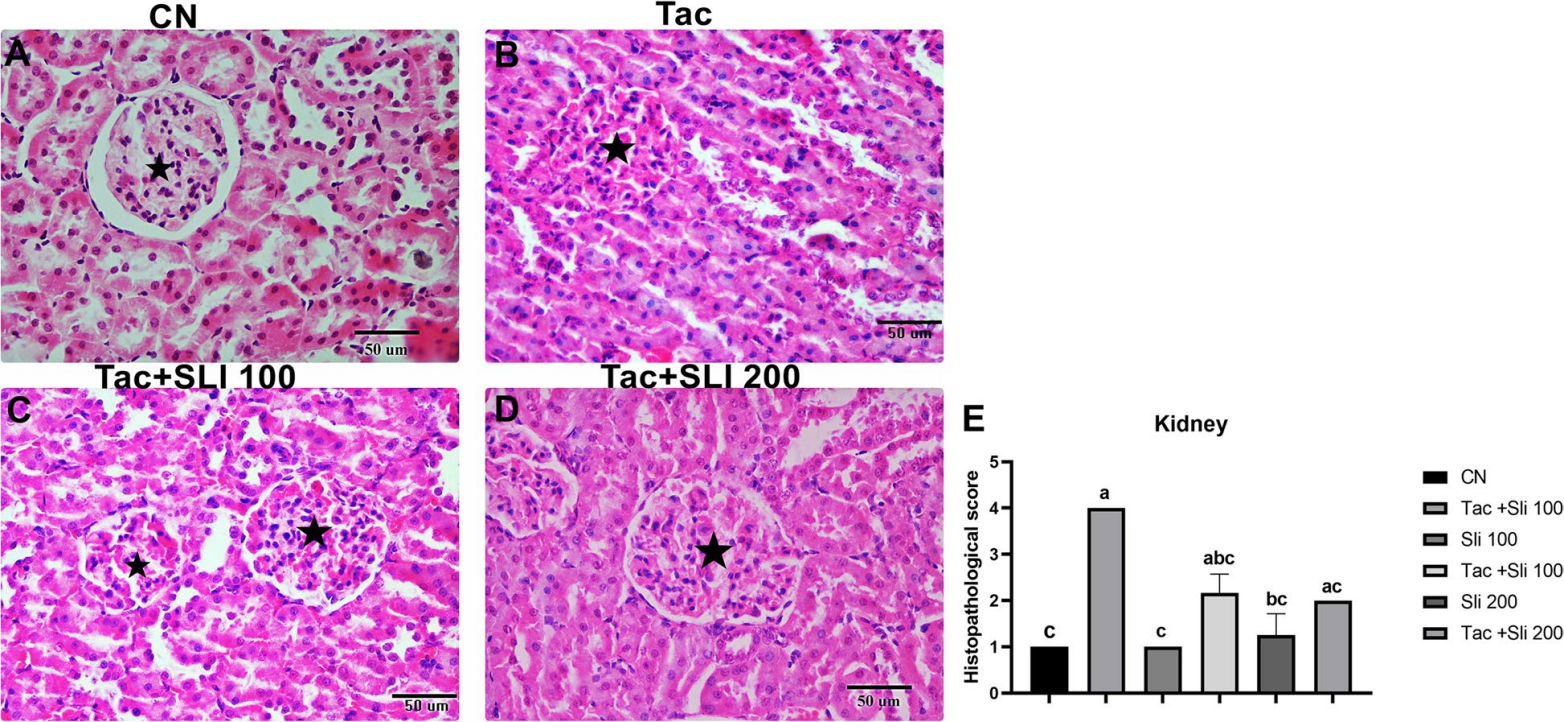
Hematoxylin-eosin staining. Kidney. **A** Glomerulus and tubules in the normal histological structure. Bar:50 μm. **B** Severe swelling in the glomeruli, hydropic degeneration, and desquamation in the tubulus epithelium (black arrows). Bar: 50 μm. **C-D** Mild swelling (stars) and hyperemia in the glomeruli, and hydropic degeneration of the tubular epithelium. Bar:50 μm. **E** Statistical expression of the kidney histopathological scores between the groups (data are presented as the mean ± standard deviation, *P* < 0.05)

**Fig. 2:**
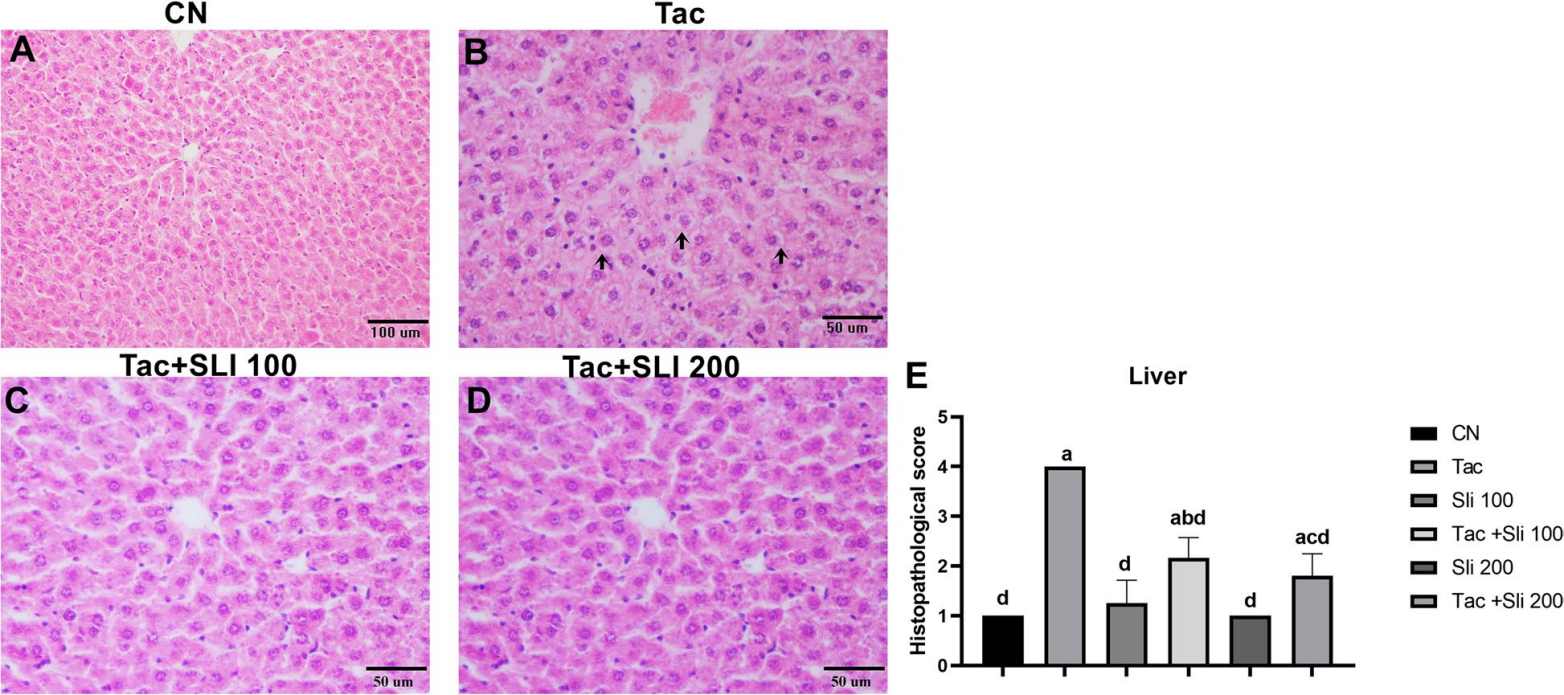
Hematoxylin-eosin staining. Liver. **A** Hepatocyte and sinusoids in the normal histological structure. Bar: 50 μm. **B** Severe hydropic degeneration and vacuole formation in the hepatocytes (arrows) and narrowing of the sinusoids. Bar: 50 μm. **C**,** D** Mild swelling in the hepatocytes. Bar:50 μm. **E.** Statistical expression of the liver histopathological scores between the groups (data are presented as the mean ± standard deviation, *P* < 0.05)

In the kidneys in the Tac group, severe swelling and hyperemia of the glomeruli, and hydropic degeneration and desquamation of in tubular epithelium, dilatation and protein rich fluid of lumens, and hyperemia of in interstitial tissue were observed (Fig. 1B). Mild swelling and hyperemia in the glomeruli and hydropic degeneration of the tubular epithelium were detected in the Tac + SLI 100 and Tac + SLI 200 groups (Fig. 1 C-D). It was not statistically significant between the CN, SLI 100, and SLI 200 groups (Fig. 1 A), while a statistically significant increase (*P* < 0.05) was determined in the Tac group (Fig. 1B-E) compared to these groups. In the hepatocytes in the Tac group, severe hydropic degeneration, vacuolization, and narrowing of the sinusoids were detected (Fig. 2B). In addition, histopathologically, Tac + SLI 100 and Tac + SLI 200 groups were found to be similar to the CN group (*P* > 0.05 Fig. 2A-C-D-E).

#### Apoptosis results

**Fig. 3:**
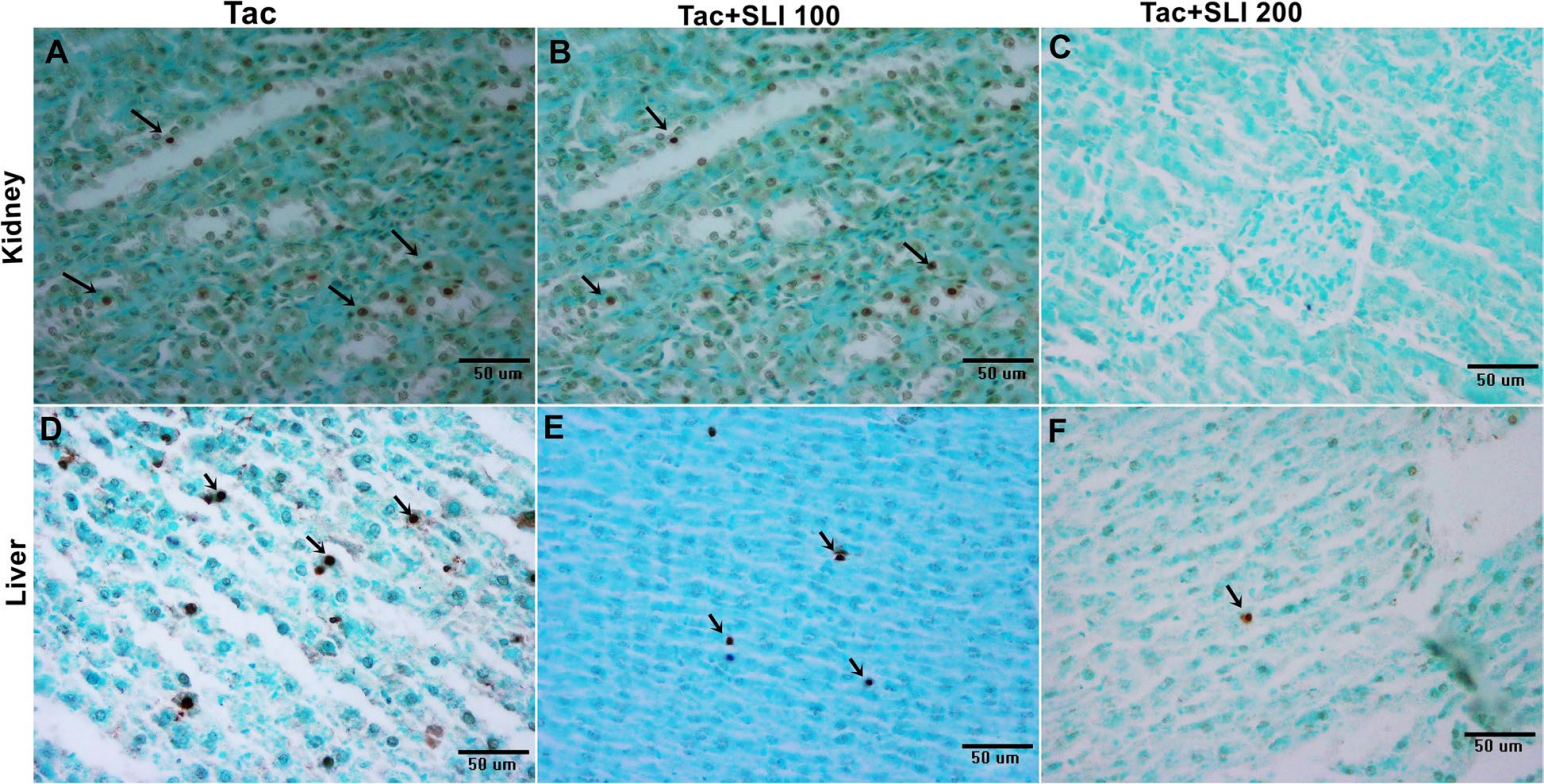
** A-B** TUNEL-positive staining in the tubulus epithelium. Kidney. **C** TUNEL-negative staining in the glomerulus and tubular epithelium. Kidney. Bar: 50 μm. **D-F** TUNEL-positive staining in the hepatocytes. Liver. Bar:50 μm

The TUNEL-positive reactions of apoptotic cells that indicate DNA breaks in the tubular epithelium and hepatocytes were the most significantly observed in the positive control preparation and the Tac group by situ TUNEL staining (Fig. 3 A-D). TUNEL-positive reactions were observed less frequently in both the kidney tubule epithelium and liver hepatocytes of the Tac + SLI 100 group (Fig. 3B-E) compared to the Tac group. In the Tac + SLI 200 group, no TUNEL-positive reactions were observed in the kidney tubule epithelium (Fig. 3C) and very few were observed in liver hepatocytes (Fig. 3F).

### Biochemical results

**Fig. 4:**
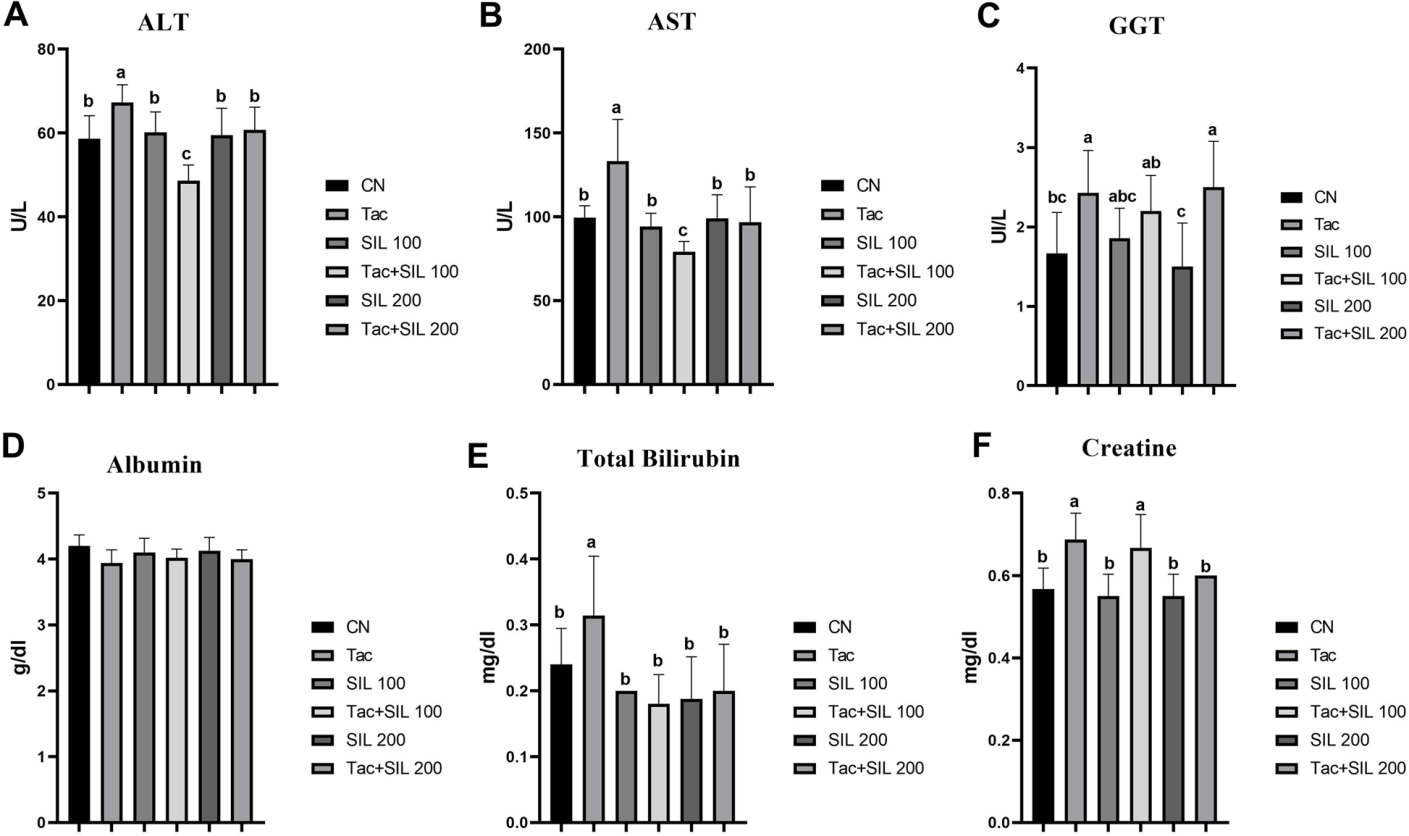
** A** Statistically significantly increased ALT level in the Tac group compared to the Tac + SLI 100 group (*P* < 0.05). **B** Significantly higher AST level in the Tac group compared to the other experimental study groups (*P* < 0.05). **C** High GGT level in the Tac and Tac + SLI 100 groups and low level in SLI 200 group (*P* < 0.05). **D** Non-statistically significant albumin level between the groups (*P* > 0.05). **E** Statistically significantly increased total bilirubin level in the Tac group (*P* < 0.05). **F** Statistically different creatine level in the Tac and Tac + SLI 100 groups compared to the other experimental groups (*P* < 0.05)

In the blood serum samples, ALT and AST levels were significantly lower in the Tac + SLI 100 group than in the Tac group (*P* < 0.05) and were statistically (*P* > 0.05) similar between the SLI 200 and Tac + SLI 200 groups (Fig. 4A-B). The GGT level was statistically higher in Tac group and Tac + SLI 200 group compared to the other study groups (*P* < 0.05) (Fig. 4C). There was no significant difference (*P* > 0.05) between study groups with regard to the albumin level (Fig. 4D). The total bilirubin level was statistically increased (*P* < 0.05) in the Tac group compared to the other study groups (Fig. 4E). The creatine level increased statistically in the Tac and Tac + SLI 100 groups (*P* < 0.05) compared to the SLI 100 and SLI 200 groups (Fig. 4F). The TOC increased significantly in the Tac group compared to the other study groups and was not statistically significant in the other groups (Table 1). The TAC level was lowest in the Tac group (2.04 ± 0.16, *P* > 0.05) and highest in the Tac + SLI 200 group (3.57 ± 0.58, *P* > 0.05).

**Table 1:** Statistical expression of TOC and TAC level in control and experimental groups

	CN	Tac	SLI 100	Tac + SLI 100	SLI 200	Tac + SLI 200
**TOC** (**µmol H**_**2**_**O**_**2**_**eq/L)**	2.33 ± 0.45^b^	11.8 ± 2.40^a^	2.93 ± 0.38^b^	5.76 ± 2.05^b^	3.19 ± 1.25^b^	4.82 ± 1.76^b^
**TAC (mmol Trolox eq/L)**	3.05 ± 0.32	2.04 ± 0.16	3.22 ± 0.27	3.03 ± 0.17	3.01 ± 0.73	3.57 ± 0.58

^a, b^ Statistical difference between the groups

*P* < 0.05

## Discussion

Tacrolimus, which is used to reduce the rejection rate in organ transplantation, causes various side effects, such as nephrotoxicity and hepatotoxicity in long-term treatment [5, 25, 47, 48]. Antioxidants are used against the side effects of immunosuppressive drugs [49, 50]. Silymarin is an antioxidant that exerts this effect by scavenging free oxygen radicals and inhibiting lipid peroxidation [33–35]. Silymarin has also been used for centuries as a therapeutic in liver and gallbladder diseases, such as hepatitis, cirrhosis, and jaundice [4, 29]. However, the effect of silymarin on tacrolimus-induced kidney and liver injuries has not been investigated. In this study, it was aimed to investigate the protective effects of silymarin against the nephrotoxicity and hepatotoxicity caused by tacrolimus by histopathological, in situ TUNEL, and biochemical methods.

The most important side effect of tacrolimus is nephrotoxicity [5, 51]. In this study, severe swelling and hyperemia were detected in the glomeruli, hydropic degeneration, necrosis, and desquamation of the tubular epithelium in rats treated with tacrolimus, similar to previous experimental studies [50–52]. Dilatation and protein-rich fluid in the tubule lumen and hyperemia were evident in the interstitial tissue. In previous studies, findings of chronic nephrotoxicity, such as interstitial fibrosis, tubular atrophy, arteriolar hyalinosis, and tubular microcalcifications were detected in kidney biopsy specimens from people treated with tacrolimus [5, 53]. These findings indicate that renal blood parameters should be monitored in both acute and chronic toxicity of tacrolimus in patients. One of the criteria used commonly for determining renal lesions is serum creatinine levels. In experimental studies, it was determined that tacrolimus increases the creatinine level [50, 54, 55]. In our study, we found that creatine level increased significantly in the Tac group.

Silymarin, a root extract obtained from *Silybum marianum*, is known to have a hepatoprotective effect against liver disease. In nephrotoxicity caused by chemotherapeutic drugs (cisplatin and cyclophosphamide) and thioacetamide, the beneficial effects of silymarin treatment on renal glomerular and tubular injuries were histologically determined [56–58]. In our study, we determined that silymarin ameliorated the histopathological changes in the glomerulus and tubular epithelium caused by Tacrolimus. In experimental animal drug nephrotoxicity studies, it was determined that silymarin was protective against renal toxicity by lowering the serum creatinine level [13, 43, 59, 60]. In this study, the creatinine level was significantly decreased in the Tac + SLI 200 group compared to the Tac group (*P* < 0.05). These findings suggest that Silymarin has a significant nephroprotective effect against tacrolimus nephrotoxicity.

In our current study, we noticed that silymarin ameliorated the histopathological changes in hepatocytes caused by tacrolimus. It was observed that liver from the rats treated with tacrolimus was pathologically altered, such as hydropic degeneration and fat vacuoles in the hepatocytes and narrowing of the sinusoids in contrast with the control group. Studies on the hepatotoxicity of tacrolimus have shown signs of chronic hepatotoxicity, such as an increase in Kupffer cells and centrilobular fibrosis, in studies on biopsy specimens [61, 62]. It is thought that antioxidants should be used to reduce the acute and chronic hepatoxic effects caused by tacrolimus. Silymarin, fungal poisoning are used therapeutically to reduce even bacterial endotoxins, viral hepatitis, and alcohol-induced liver damage [28]. However, no studies on the protective effects of silymarin against tacrolimus hepatotoxicity could be found. In our study, we determined that tacrolimus significantly increased ALT, AST, GGT, and bilirubin, while silymarin reversed it. Liver function tests, such as ALT, AST, and GGT, are most commonly used to determine liver damage. An increase in the ALT level indicates mild liver damage, an increase in the AST level indicates cell necrosis, and an increase in the GGT and bilirubin levels indicates liver failure [63]. Silymarin has a hepatoprotective effect against paracetamol, carbon tetrachloride and diclofenac-induced liver toxicity by reducing AST and ALT levels [37–39]. In our study, we determined that Tacrolimus significantly increased ALT, AST, GGT, and bilirubin, while silymarin reversed it. In this study, no statistically significant difference was found between the other study groups in the albumin level in the Tac group (*P* > 0.05). However, the lower albumin level in the Tac group compared to the other groups indicated the presence of hepatocellular degeneration. The findings of our study show that silymarin has a protective hepatoprotective effect against the hepatotoxicity of tacrolimus.

In our study, we found that tacrolimus caused oxidative stress. Oxidative stress consists of an imbalance between cellular ROS. Immunosuppressive agents cause changes in cells and tissues by promoting the formation of free oxygen radicals, such as hydroxyl radicals (OH¯), superoxide anions (O2¯), and peroxyl radicals (ROO¯) [64]. Measurement of these oxidant molecules individually is impractical, instead, total oxidant capacity (TOC) may be preferred. In the case of oxidative stress, antioxidants such as catalase, glutathione (GSH), glutathione peroxidase (GSH-Px) and superoxide dismutase (SOD) are consumed in the cells. Antioxidant parameters such as peroxide dismutase (SOD) and thioredoxin (TRX) catalase and glutathione were decreased in tacrolimus-treated animals [50, 65]. It is difficult to measure each antioxidant parameter to determine the antioxidant status; thus, the total antioxidant capacity (TAC) was determined in this study. In previous studies [66, 67], it was determined that TOC increased in rats with renal ischemia-reperfusion injury (I-R) and ischemia-reperfusion injury in the abdominal aorta, and TAC increased in silymarin treatment. In our study, we noticed that while TOC levels increased in rats treated with Tacrolimus, silymarin decreased it (*P* < 0.05). In addition, it was observed that TAC level decreased in tacrolimus applied rats and increased in silymarin applied groups (*P* > 0.05). In experimental studies, TAC and TOC measurements can be preferred as a predictor for determining oxidative stress and antioxidant effects.

In this study, a large number of TUNEL-positive reactions were detected in tacrolimus-treated rat kidney and liver cells, indicating DNA fragmentation. Although many ways exist to activate caspases, only two are known in detail. Tacrolimus causes nuclear fragmentation and apoptosis with bak protein expression, mitochondrial dysfunction and caspase-3 activation [68, 69]. In an experimental study, it was shown that tacrolimus induces apoptosis in endothelial cells of capillaries in the brain and causes a partial encephalopathy [70]. In addition, in this study, fewer apoptotic cells were detected in the liver and kidney tissue in the silymarin-treated groups compared to the Tac group. It has been reported that methotrexate and colistin cause apoptosis in kidneys, fumonisin-B in liver hepatocytes, and silymarin has antiapoptotic effects [44, 71, 72]. In our study, it was determined that tacrolimus caused apoptosis in the kidney and liver and silymarin contributed to the prevention of apoptosis.

Numerous phase I clinical trials have been conducted on CYP enzymes, mainly due to silymarin/silybin-induced herb-drug interactions [73, 74]. Oral administration of silybin to rats showed that tamoxifen (P-gp, CYP3A4 substrate) could significantly alter the pharmacokinetics [75]. Silybin has been reported to inactivate recombinant cytochrome P450 3A4 and 2C9 [76]. In addition, careful administration of silybin with drugs cleared by P450 3A4 or 2C9 has been indicated. Tacrolimus is also metabolized by CYP3A4 isoenzymes and P-glycoprotein in the intestinal mucosa, followed by CYP3A4 (and to a lesser extent CYP3A5) isoenzymes in the liver [10, 11]. In an experimental study in rats, it was noted that silymarin did not have a significant effect on the pharmacokinetic parameters of FK506 (tacrolimus) [77]. Our approach has some limitations. First, the herb-drug pharmacokinetics of silymarin and tacrolimus, such as the actions of cytochrome P450 (CYP) enzymes, UDP-glucuronosyltransferase (UGT) enzyme activity, and P-glycoprotein (P-gp) transport, have not been investigated. In vivo and in vitro pharmacokinetic studies should be performed to determine the herb-drug interactions of tacrolimus and silymarin. Secondly, we could not perform the blood concentration of tacrolimus in rats. Determining the blood concentration of tacrolimus after tissue transplantation in humans is important for determining the effective dose of tacrolimus.Previous studies [23, 68] reported toxic doses of tacrolimus in rats. In our study, we histopathologically and biochemically determined nephrotoxic and hepatotoxic effects at a tacrolimus dosage of 1 mg/kg/day for 6 weeks.

## Conclusions

In our study, silymarin was co-administered with tacrolimus to minimize the renal and hepatic toxic effects of tacrolimus in the rat model at the chosen dosage levels. The histopathological, biochemical, and apoptotic findings suggested that silymarin has a protective effect against the nephrotoxicity and hepatotoxicity caused by tacrolimus at the doses administered here. It was previously reported that patients with a histological diagnosis of calcineurin inhibitor nephrotoxicity had lower rates of graft failure than those without such a diagnosis [78]. Considering the long-term use of tacrolimus and other immunosuppressive drugs in cases of organ transplantation, more long-term experimental studies are needed to clarify the chronic side effects and nephrotoxicity of tacrolimus and the protective effects of silymarin.

## Data Availability

The datasets used and/or analysed during the current study are available from the corresponding author on reasonable request.
